# Three-dimensional measurement of foot arch in preschool children

**DOI:** 10.1186/1475-925X-11-76

**Published:** 2012-09-25

**Authors:** Hsun-Wen Chang, Chien-Ju Lin, Li-Chieh Kuo, Ming-June Tsai, Hsiao-Feng Chieh, Fong-Chin Su

**Affiliations:** 1 Department of Biomedical Engineering, National Cheng Kung University, 1 University Road, Tainan, 701, Taiwan; 2 Department of Physical Therapy, Fooyin University, 151 Jinxue Rd., Daliao Dist, Kaohsiung City, 83102, Taiwan; 3 Medical Device Innovation Center, National Cheng Kung University, 1 University Road, Tainan, 701, Taiwan; 4 Department of Occupational Therapy, National Cheng Kung University, 1 University Road, Tainan, 701, Taiwan; 5 Department of Mechanical Engineering, National Cheng Kung University, 1 University Road, Tainan, 701, Taiwan; 6 Department of Materials Science and Engineering, National Cheng Kung University, 1 University Road, Tainan, 701, Taiwan

**Keywords:** Foot, Flatfoot, Preschool children, Three-dimensional image, Anthropometry

## Abstract

**Background:**

The prevalence of flexible flatfoot is high among preschool-aged children, but the effects of treatment are inconclusive due to the unclear definitions of normal flatfoot. To date, a universally accepted evaluation method of the foot arch in children has not been completely established. Our aims of this study were to establish a new method to evaluate the foot arch from a three dimensional perspective and to investigate the flexibility of the foot arch among children aged from two to six.

**Methods:**

A total of 44 children aged from two to six years of age were put into five age groups in this study. The navicular height was measured with one leg standing, and both feet were scanned separately in both sitting and one leg standing positions to compute the foot arch volume. The arch volume index, which represents the ratio of the difference in volume between sitting and one leg standing positions to the volume when sitting was calculated to demonstrate the flexibility of the foot arch. The differences of measured parameters between each aged group were analyzed by one-way ANOVA.

**Results:**

The arch volumes when sitting and standing were highly correlated with the navicular height. The navicular height ranged from 15.75 to 27 mm, the arch volume when sitting ranged from 6,223 to 11,630 mm^3^, and the arch volume when standing from 3,111 to 7,848 mm^3^ from two to six years of age. The arch volume index showed a declining trend as age increased.

**Conclusion:**

This study is the first to describe the foot arch with volume perspective in preschool-aged children. The foot arch volume was highly correlated with the navicular height. Research results show both navicular height index and arch volume index gradually increase with age from two to six. At the same time the arch also becomes rigid with age from two to six. These results could be applied for clinical evaluation of the foot arch and post-treatment evaluation.

## Background

Flexible flatfoot is one of the most common conditions seen in pediatric orthopedic clinics. The foot arch begins to develop when a child starts to take weight on their legs, and it keeps developing during the first decade of life. The prevalence of flexible flatfoot is 21-57% in children at preschool age [1,2]. Although the prevalence decreases with age, flatfoot may lead to further abnormalities and cause pain or influence the performance of physical tasks and walking [2,3]. According to Lin’s ([2001]) [2] and D’Amico’s ([1984]) [4] studies, children with flatfoot performed physical tasks poorly and it may cause gait disorders in future. Flatfoot should not only be considered as an abnormal ankle and foot complex, but also as a reference of gross motor development, and more precise periodic assessments of the foot arch could reveal more about this issue.

Pediatric flatfoot is the flattening of the medial longitudinal arch (MLA) of the foot [2]. There are two types of flatfoot: flexible flatfoot and rigid flatfoot. A flexible flatfoot is defined as collapsing of the foot arch when weight is put onto the foot during standing or walking. A rigid flatfoot is defined as a permanently fixed deformation in the flat position no matter with weight bearing or not. The MLA allows the foot to transfer weight and absorb shock in the erect posture [5]. Footprints, radiographs, and anthropometric measures are used to measure the MLA [1,6,7]. However, the validity and reliability of these methods are in dispute [6,8-15]. While the majority of flatfoot measurements acquire two-dimensional (2D) information about the foot, the term “flatfoot” encompasses multi-site three-dimensional (3D) deformities, including hindfoot pronation, subtalar joint dorsiflexion and external rotation, the midfoot abduction, and the forefoot supination in relation to the hindfoot. A 2D assessment is thus not able to supply comprehensive information about the MLA, and it is believed that 3D data would provide the most accurate evaluation of the foot arch. 3D laser scanner technology is wildly applied in various fields [16-19]. Recently, a 3D scanner was used to measure foot behavior [16,20,21], although only 2D parameters were discussed, including the forefoot varus angles and foot dimensions, and only a few studies have used 3D parameters to describe the foot arch, and thus establishing 3D parameters to assess the foot arch is a critical issue.

The foot plays an important role in maintaining a static position and providing a stable base when performing functional activities. However, it remains as open question as to whether the static appearance of the foot arch can predict dynamic foot arch behavior. Some studies supported that static foot arch measurement was in significant correlation with dynamic measurement system [22-24]. Some studies concluded the opposite results. Trisha et al. proposed that a static measure of the MLA could not predict the dynamic motion of the MLA [25], while Cavanagh et al. stated that only limited variance in dynamic plantar pressure can be explained by such measurements [26]. Therefore an index to describe the flexibility of the MLA is required.

The foot dimensions and shapes have large variations in children due to the various stages of their development, making it more difficult to establish what a normal foot arch is in children. Without a normal range of the MLA, a clinician might have difficulties in making correct diagnostic as well as intervention decisions. The objective assessment of the characteristics of MLA in children of different age groups would thus aid clinical professionals in identifying children with flatfoot, observing children’s development, and comparing the effect of intervention. To establish a database needs a large number of samples. In this study, we tried to build a reference range of each age group from our limited recruited samples. Next we can further establish a database based on the result of this study in future project. The purpose of this study was thus first to establish a 3D parameter of foot arch to evaluate preschool-aged children, and secondly to describe the flexibility of the foot arch from 3D perspectives. In future, we will establish a normal database of the 3D parameter of foot arch in preschool-age children.

## Methods

### Subjects

Forty-four children (24 boys, 20 girls) without foot problems were recruited from kindergartens in southern Taiwan. The children were put into five age groups from two to six years old. The characteristics of the subjects for each age group are shown in Table 1, and there are some significant differences between the groups. Subjects were excluded if the following conditions were presented: (i) diagnosis of fixed foot deformity, (ii) pain in the ankle or foot within the last three months, or (iii) evidence of developmental disabilities that may influence the development of the foot. Consent was obtained from all the parents of the children before undergoing the test procedure, and this study was approved by the Institutional Review Board of Fooyin University Hospital (FYH-IRB-099-06-03).

**Table 1:** The means and standard deviations of the characteristics of the subjects

**Age group**	**N (feet)**	**Age**^**c**^**(month)**	**Height**^**c**^**(cm)**	**Weight**^**c**^**(kg)**	**CMFW**^**ac**^**(cm)**	**CMFL**^**bc**^**(cm)**
2	16	31 ± 3.50	87.94 ± 4.78	11.71 ± 1.49	6.08 ± 0.39	14.10 ± 0.82
3	14	43 ± 2.47	99.61 ± 4.27	15.11 ± 2.32	6.70 ± 0.45	15.88 ± 1.31
4	18	56 ± 2.95	108.00 ± 4.00	18.28 ± 2.11	7.11 ± 3.91	17.29 ± 0.76
5	18	69 ± 3.4	112.36 ± 4.89	20.61 ± 3.52	7.43 ± 0.35	18.43 ± 0.84
6	22	76 ± 2.42	117.51 ± 3.63	22.64 ± 3.62	7.47 ± 0.39	18.47 ± 0.96

^a^CMFW: clinical measuring foot width; ^b^CMFL: clinical measuring foot length.

^c^a significant difference between different ages (*p* < 0.05).

### Equipment

**Figure 1:**
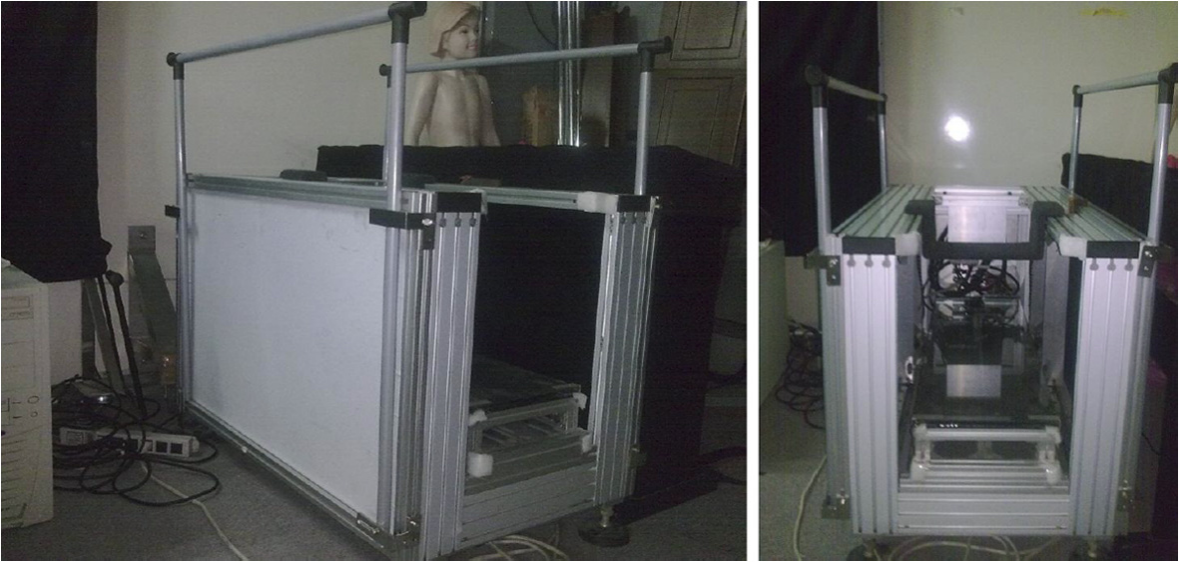
The schematic picture of the “Peripher 3D Scanner” from different views.

The 3D foot contour was scanned using “Peripher 3D Scanner” (Figure 1) made by the Robotic and Automation Research Laboratory at National Cheng Kung University in Taiwan. The system is composed of a measuring system and an image processing unit, with a 32-bit personal computer as a controller. The measuring system has four scanning modules, and each module includes two coupled charged devised (CCD) cameras, one sliced-ray laser projector and two mirrors. The laser projector projects a stripe of light on the object to be measured, and the deformation of the stripes, based on the object's topography, is captured by the CCD cameras aimed at the mirror. These deformed light stripes are captured by the CCD cameras at 1 mm intervals until the total scanning procedure is finished and digitized by the image acquisition card and stored in the main memory of the personal computer. A foot can be modeled using many “slices” of scanned data obtained along the length of the foot.

### Procedure

#### Clinical anthropometric measurements

**Figure 2:**
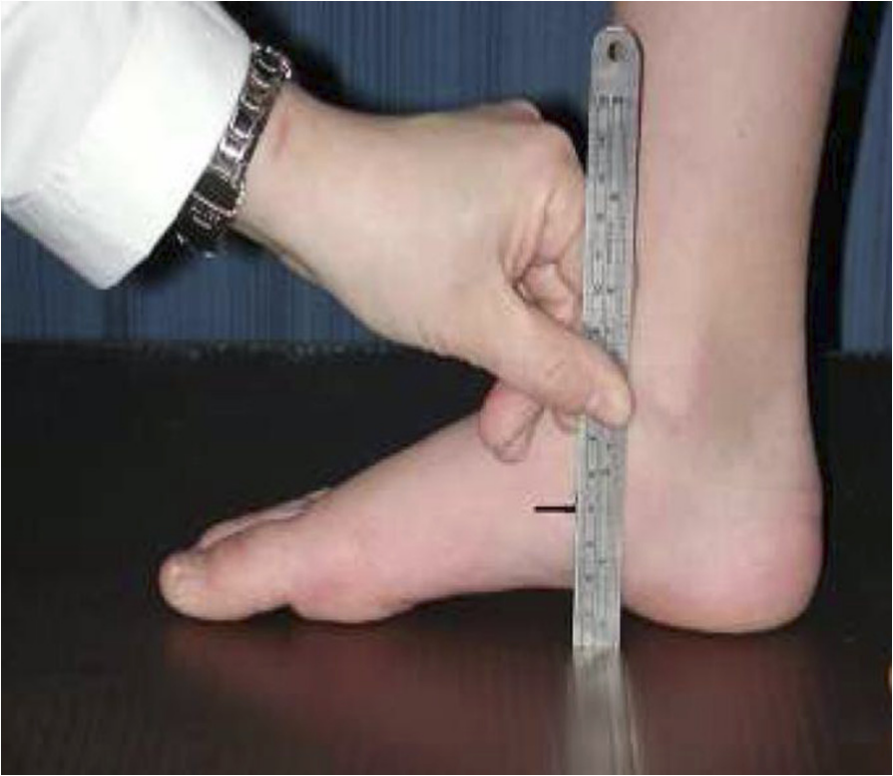
The measurement of navicular height.

The subject was standing on one leg on an elevated platform with the other leg supported on a stool. The width and length of both feet were measured with a Metrology digital caliper (Taiwan) with a resolution of 0.01 mm. The foot width was measured between the first and fifth metatarsal heads. The foot length was measured from the most posterior point of the calcaneus to the end of the longest toe. The navicular height (NH) (Figure 2) was obtained from the lowest palpable medial projection of the navicular to the supporting surface. All the measurements were performed by the same experienced physical therapist. A set of data, including twenty feet with the NH measured twice by the same physical therapist, was used to evaluate the intraobserver reliability. The ICC (3, 1) was 0.89, representing high reliability between different measurements.

#### Foot scanning

**Figure 3:**
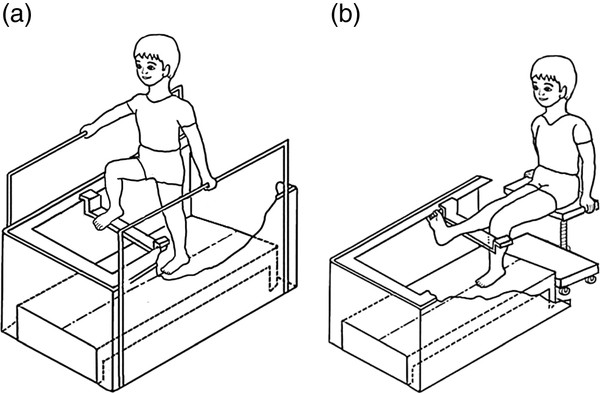
The testing postures of the subject in the 3D scanner. (a) standing, and (b) sitting.

Each foot was scanned by the “Peripher 3D Scanner” three times in each of the two positions: sitting and standing. In the sitting position, the subject was positioned on a height adjustable seat and kept the hip and knee joints at 90° of flexion and the ankle joint in a neutral position. In the standing position, the subject was asked to stand on one leg and permitted to hold onto a rail to maintain their balance. The standing posture would be kept with all the following landmarks were in alignment which include acromion process, the hip center, the knee joint center and the lateral malleolus. The scanned foot was positioned with the long axis of the foot aligned on the axis of the platform, while the other foot was suspended in front (Figure 3). It would take 15 s for scanning one foot.

#### Data processing

A commercial software, Geomagic Studio 10, was utilized to reconstruct a 3D model of each foot. The landmarks of the 3D image of the foot were digitized by the same researcher. The outline of the MLA, including the landmarks of the center of the midfoot, the first metatarsal head, and the medial point on the half of the heel, was digitized, and these points created a plane called the arch plane. The arch plane projecting to the supporting surface then formed the volume of the foot arch. The foot length and width and the volume of the foot arch were calculated using Geomagic Studio 10. An arch volume index (AVI) [20] was calculated by representing the flexibility of the MLA during nonweight bearing and full weight bearing situations.

$$\text{AVI}=\frac{V_{n}-V_{f}}{V_{n}}$$

V_n_ and V_f_ represent the volume under the foot arch in a sitting and standing position, respectively.

A subset of 10 feet was used to determine intraobserver reliability. The intraobserver reliability established by the ICC (2, 1) for processing the arch volume, foot width and foot length were 0.952, 0.935, and 0.988, respectively.

### Statistical analysis

Statistical analysis was performed using SPSS 17 software. A significance level of a *p*-value less than 0.05 was used for all the analysis. The mean of the subject’s demographic data was calculated. The correlations between clinical manual measured parameters and 3D scanned parameters were analyzed using Pearson correlation coefficients. The differences between these two methods were analyzed with the paired *t* test. The differences of the parameters between different age groups were analyzed by one-way ANOVA. A power analysis [27] was used to determine the required number of subjects (α = 0.05, β = 0.20) to understand the differences between each age group. The suggested minimum sample number to detect significant change is 65, and we had tested 88 samples.

## Results

The Pearson’s correlation coefficients are shown in Table 2 to better understand the relationship between the parameters measured by manual measurement and 3D scanning. Significant correlations exist between both sets of parameters. The statistical results demonstrate high correlations for both foot width (*r* = 0.859) and foot length (*r* = 0.962) between the manual and scanning measures. The NH is moderately to highly correlated with the index of foot volume in both sitting and standing (*r* = 0.642 and 0.712, respectively).

**Table 2:** **The correlation of clinical measurement with the 3D scan of the foot** (n = 88)

	**3D scanning parameters**			
	**FW**^**b**^	**FL**^**c**^	**V**_**n**_^**e**^	**V**_**f**_^**f**^
CM^a^ FW^b^	0.859^g^			
CM^a^ FL^c^		0.962^g^		
CM^a^ NH^d^			0.642^g^	0.712^g^

^a^CM: clinical measuring; ^b^FW: foot width; ^c^FL: foot length; ^d^NH: navicular height; ^e^V_n_: the volume under foot arch in a sitting position; ^f^V_f_: the volume under foot arch in a standing position; ^g^correlation is significant at 0.01 level.

**Figure 4:**
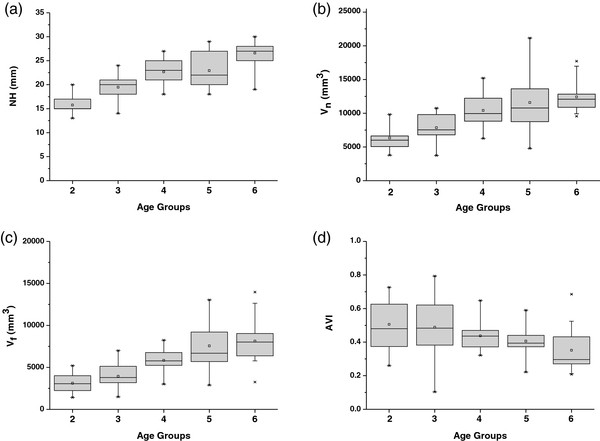
**Box plot of the NH (a), V**_**n**_**(b), V**_**f**_**(c), and AVI (d) in groups from ages two to six.** The box includes observations from the 25^th^ to the 75^th^ percentile; the horizontal line within the box represents the median value, and the square represents the mean value. Lines outside the box represent the 1^th^ and 99^th^ percentiles. NH: navicular height V_n_: the volume under foot arch in a sitting position V_f_: the volume under foot arch in a standing position AVI: arch volume index.

The 25% to 75% of the normal range of NH, V_n_, V_f_ and AVI for children are shown using a box figure in Figure 4 in order to provide a reference to distinguish flatfoot at ages two to six. According to the report from Cavanagh et al. (1987) [28], the approach of using ± 1 S.D. to delineate the “normal” arch would result in only 15% of the sample being placed in each of two extreme groups. However, the prevalence of flatfoot is higher than 15% in children at preschool age according to the previous reports [1,2]. Thus the approach of using quartile [28] in this study seems more appropriate to delineate the “normal” arch. Therefore, 1^st^ quartile of the measured range of the arch parameters is used to define flat foot in this study.

**Figure 5:**
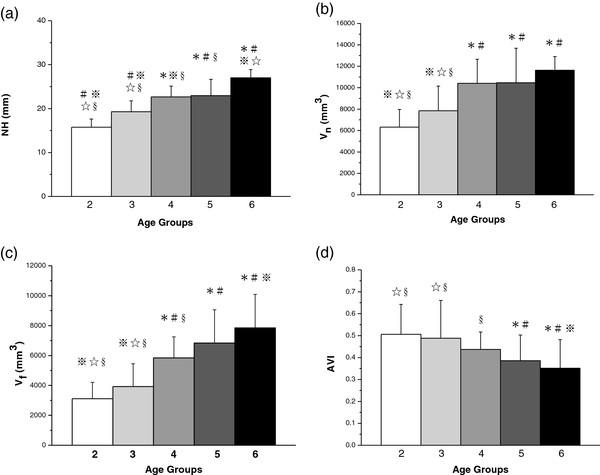
**The comparisons of the NH (a), V**_**n**_**(b), V**_**f**_**(c), and AVI (d) for five groups.** (* vs. age two; # vs. age three; ※ vs. age four; ☆ vs. age five; § vs. age six; *p* < 0.05).

The means and standard deviations of the NH for different age groups are shown in Figure 5(a). The means of the NH are 15.75 mm, 19.29 mm, 22.67 mm, 22.94 mm and 27.00 mm in order of ages two to six. The NH increases 3-4 mm every year from age two through age six, except from ages four to five. The results of one-way ANOVA show a significant effect of age on NH (*p* < 0.0001). Post-hoc LSD tests indicate that the NH is significantly different between each age group, except for the groups aged four and five.

The means and standard deviations of the V_n_ are presented in Figure 5(b). The means of the V_n_ for different age groups from two to six are 6,223, 7,846, 10,415, 10,457, and 11,630 mm^3^, respectively. The values are similar at four and five years of age. A significant difference is found in V_n_ between different groups (*p* < 0.0001). The V_n_ of the aged four, five and six groups are significantly larger than those of the aged two and three groups. However there is no significant difference among the groups aged four, five and six, and between those aged two and three.

The measurements of V_f_ are shown in Figure 5(c). The means of the V_f_ for different age groups from two to six are 3,111, 3,926, 5,845, 6,832, and 7848 mm^3^, respectively. V_f_ thus rises along with age. To analyze the age factor, the results of the one-way ANOVA indicate significant differences in V_f_. Post-hoc LSD tests indicate that the V_f_ is significantly different between each age group, except between the aged four and five groups. The V_f_ for the groups aged four, five and six are significantly larger than those for the aged two and three groups. There is no difference between the groups aged two and three or between those aged four and five. The V_n_ and V_f_ show similar results, that there is limited changed from age four to five, with the most significant increase occurring in the groups aged three and four.

The AVI representing the flexibility of the MLA shows the opposite tendency to the other parameters, decreasing with increasing age. In Figure 5(d), the means of the AVI of each age group, from youngest to oldest, are 0.51, 0.49, 0.43, 0.39, and 0.35. The results of one-way ANOVA show a significant effect of age on AVI (*p* < 0.01), indicating that the MLA becomes more rigid with increasing age.

## Discussion

There are no consistently clinical criteria for defining flatfoot, and this makes it difficult for the clinician to plan a precise treatment protocol. The root of problem plight is the lack of a universally accepted definition of a “normal” longitudinal arch. The traditional clinical measurements of arch height typically describe the vertical height of some bony landmarks of the foot with respect to the horizontal surface. However, this measurement only considers the height of the foot arch and neglects the other two dimensions. Meanwhile, the variations of the bone make it difficult for clinicians to accurately identify the landmarks, raising concerns about reliability. With regard to the anatomic structure of the MLA, the development of 3D detection of the foot shape would be able to describe changes in position of single structural elements of the foot, which 2D measures are not able to completely describe. This technology will aid in uncovering explanations of the causes of the biomechanical reactions of structural elements in the foot [29]. In this study, we define the foot arch with volume using a 3D scanner with good accuracy to demonstrate the range of the foot arch for preschool children from a 3D perspective.

### Theoretical and practical implication

The findings of this study indicate a high correlation in foot length and width between manual measures and those obtained with a 3D scanner. These results provide support for the use of a 3D scanner, and demonstrate that it is a valuable instrument to measure the dimensions of the foot. The correlation between NH from the manual measures and V_n_ and V_f_ from the 3D scanner measures is moderate to high (*r* = 0.642, 0.712; *p* < 0.01). This indicates that V_n_ and V_f_ are able to reflect the vertical height of the MLA. V_n_ and V_f_ provide more information, not only about the vertical height, but also about the width and the length of the MLA. Therefore, the volume index can accurately describe the appearance of the MLA.

The range of normal values of the NH, V_n_ and V_f_ are reported in this study, and these parameters increase from the ages two to six. These results indicate that the development of the MLA is progressive with increasing age during the preschool years. The progressive increase of the MLA confirms the findings of previous studies [30-32]. For example, Staheli et al. indicated that the arch increased significantly from ages two to six [31], while Henning concluded that the foot arch matured at the age of six [30]. In addition, a rapid progression of the MLA occurring between two and six years old was reported by Volpon in 1994 [32]. However, Gould et al. reported that while arch development was faster during the first two years after starting to walk, it continued until five years of age [33]. In previous studies there are no databases for the normal range of the NH for children aged two to six. In the current study, the normal range of NH for children is reported, and the results show that it increases with age. The NH of the group aged six is 27 mm, which agrees with the finding of a previous study which measured the NH from ages six to 10 [12]. V_n_ and V_f_ separately indicate the foot arch volume in different weight bearing situations. V_n_ is the foot arch volume under non-weight bearing conditions, and it represents the condition of the bony structure of the foot. V_f_ is the foot arch volume under full-weight bearing, and represents the condition of the soft tissue and bony structure of the foot. These parameters can identify different problems. For subjects who are unable to stand, such as those with severe cerebral palsy, the V_n_ detected in the sitting position can be used to identify foot arch problems. The range of normal values of the NH and V_n_ and V_f_ for each age group is suggested for use as an indicator to identify abnormally low arches.

The changes of the NH, V_n_ and V_f_ are not significant at ages four to five. Leung et al. studied the development of foot arch function measured by the contact force ratio (CFR) of Chinese children from ages four to 18 [34]. The results showed that the CFRs were similar at four and five, which agrees with our findings. The temporarily paused development of MLA at ages four and five may be due to several reasons. The first is that the woven bone, which is formed during the fetal period and is more flexible, has converted to the lamellar bone, which becomes harder by four years of age [35]. The second one is that the ossification of the sustebtaculum tali begins at approximately four or five years of age, but is not complete for at least another one to two years [32]. However, Onodera used different indexes, measured from footprints, to evaluate the MLA, and concluded that there was significant difference in MLA between four and five years old [36], which may be due to the different evaluation methods. This study found significant increases in V_n_ and V_f_ at ages three to four. This is supported by Gould, who concluded that arch development was fastest during the first two years after starting to walk [33]. In Figure 5, NH seems to have a higher discrimination rate between each age category. However, NH is an index which only represents the height of the medial longitudinal arch and neglects the other two dimensions. Therefore, it not really reflects the completed characteristics of the arch. The discrimination rate shown in the Figure 5 (a) may not really reflect the difference of the arch between each age group. Volume indexes show the volume under the medial longitudinal arch which consider not only the height but also the length and width of the arch. It is an index which considers in 3D dimension of the arch than NH does. Although the volume indexes show lower discrimination rate than NH, they may actually represent a correct description on the development of the foot arch. Basmajian et al. reported that the support of the MLA is ligamentous, and that the muscle is used only as a dynamic stabilizer [37]. However, there are few studies that investigate the biomechanical properties of the ligaments at different ages in growing children, and this could thus be a topic for future research.

The value of V_f_ is smaller than that of V_n_, indicating that the MLA is not a completely rigid structure before the age of six, and that it will deform depending on the loading of the leg. The difference between V_n_ and V_f_ is represented by the AVI, which is able to indicate the dynamic change of the MLA between lower extremity with and without weight bearing. Although the V_n_ and V_f_ increase from ages two to six, the AVI decreases with age, which means that the MLA becomes more rigid over time. V_n_ and V_f_ only provid information about foot arch in a static condition. However the flexibility of flatfoot is a more important feature than the static shape. Jack proposed the “toe-raising test” to identify the flexibility of the foot arch by dorsiflexion of the toes to shorten the distance between the calcaneus and metatarsals and elevate the MLA [38]. While this method is widely used in clinics to evaluate the flexibility of the foot arches, it is a subjective method depending on the evaluator, and does not provide quantitative information. Navicular drop test [39] was proposed by Brody, 1982. The navicular tuberosity is measured on the non-weight bearing foot with the subtalar joint in neutral position. Then the navicular height is measured in standing position with 50% weight bearing on the foot. The difference of the navicular height between sitting and 50% weight bearing was indicated the dynamic performance of the foot arch. Moderate interrater and intrarater reliability was reported by Picciano et al. [40] Poor reliability may be attributed to subjective judgment of subtalar joint position and 50% weight bearing. By the way, navicular drop only represents the absolute height difference of the navicular height between non-weight bearing and 50% weight bearing. I will suggest that navicular drop should be normalized by its original height as further approach. In contrast, the AVI is an objective index measured with an accurate instrument that is able to quantify the flexibility of the foot arch. Considering both the V_f_ index and AVI will help us to differentiate between high and low arch, rigid and flexible of the arch. Definition the rigid flat foot children with an V_f_ value in 1^st^ quartile of the measured range indicate as a flatfoot; meanwhile, AVI values in 1^st^ quartile of the measured range are considered as having rigid flatfoot and AVI values beyond 1^st^ quartile are considered as having a flexible flatfoot.

### Study limitations

Establishing a database of foot arch is important to make a correct assessment. In this study, typical developmental children were recruited only and which were not able to represent the general preschool children with limited sample number. However, we develop a new tool to evaluate the foot arch with a three dimensional perspective and establish the foot arch volume for the typical developmental preschool children as reference. We will recruit more samples including deformed foot to establish a database of foot arch volume using this three dimensional model in future study.

## Conclusions

A 3D laser scanner is a fast and easily operated system that can provide accurate 2D foot dimensions but also estimate 3D arch volume. It could be applied to clinical evaluation and also the post-treatment evaluation. This study establishes the range of the volume indexes for typical developmental children from age two to six. The study results might be a extremely valuable reference for clinical screening of the foot arch to decide an accurate treatment protocol. The AVI can also be used as an index to distinguish the type of flatfoot. The volume index which is generally associated with the shape of the foot may be more appropriate to represent the foot arch function and abnormalities than traditional 2D foot arch parameters. 3D parameters can be applied to the clinical evaluation of the foot arch to establish a database of the foot arch in future.

## Competing interests

The authors declare that they have no competing interests.

## Authors' contributions

HWC participated in the research design, data collection, data analysis, data interpretation, and manuscript writing. CJL participated in the research design, data interpretation, and manuscript revision. LCK participated in the data interpretation, and manuscript revision. MJT participated in the instrumental setup. HFC participated in the research design. FCS participated in the experimental setup and final approval of the version. All authors read and approved the final manuscript.
